# Hybrid Anomaly Detection in Time Series by Combining Kalman Filters and Machine Learning Models

**DOI:** 10.3390/s24092895

**Published:** 2024-05-01

**Authors:** Andreas Puder, Moritz Zink, Luca Seidel, Eric Sax

**Affiliations:** 1 Embedded Systems, Getinge AB, 76437 Rastatt, Germany; 2 Institute for Information Processing Technologies (ITIV), Karlsruhe Institute of Technology (KIT), 76131 Karlsruhe, Germany; moritz.zink@kit.edu (M.Z.); luca.seidel@kit.edu (L.S.)

**Keywords:** medical, machine learning, deep learning, anomaly detection, sensor fusion, time series analysis, Kalman filter, service-oriented architecture (SOA), ROS2, simulation

## Abstract

Due to connectivity and automation trends, the medical device industry is experiencing increased demand for safety and security mechanisms. Anomaly detection has proven to be a valuable approach for ensuring safety and security in other industries, such as automotive or IT. Medical devices must operate across a wide range of values due to variations in patient anthropometric data, making anomaly detection based on a simple threshold for signal deviations impractical. For example, surgical robots directly contacting the patient’s tissue require precise sensor data. However, since the deformation of the patient’s body during interaction or movement is highly dependent on body mass, it is impossible to define a single threshold for implausible sensor data that applies to all patients. This also involves statistical methods, such as Z-score, that consider standard deviation. Even pure machine learning algorithms cannot be expected to provide the required accuracy simply due to the lack of available training data. This paper proposes using hybrid filters by combining dynamic system models based on expert knowledge and data-based models for anomaly detection in an operating room scenario. This approach can improve detection performance and explainability while reducing the computing resources needed on embedded devices, enabling a distributed approach to anomaly detection.

## 1. Introduction

Challenges such as the aging population [1] and the increasing prevalence of chronic diseases [2] are putting pressure on healthcare systems worldwide. A strong correlation between an aging population and an increase in the incidence and mortality of chronic diseases like cardiovascular diseases, tumors, and diabetes [3] exists. Thus, there is a need to increase efficiency in all areas of healthcare, including Operating Room (OR). Medical devices that control physical objects while constantly interacting with other connected devices are considered to facilitate effective and affordable healthcare [4]. Consequently, connectivity and automation in the OR promise to contribute to solving these challenges, for instance, by the increased application of robotic medical devices. In addition, Service-oriented Device Connectivity (SDC) [5], which is a family of medical device communication standards, intends to enable data exchange and manufacturer-independent interoperability of medical devices in the OR to improve patient care.

Nonetheless, this increased connectivity also increases the risk of potential cyberattacks on these devices [6]. Historically, medical devices have operated in isolation without permanent connections to external networks, like the Internet [7]. These devices have been typically self-contained, embedded systems. Therefore, security requirements were not in scope during development, and manufacturers focused on safety. By opening these devices up to connectivity with other devices, new safety risks to patient health arise from security threats.

Operating Room Tables (OR tables) are robotic medical devices that will leverage the potential of connectivity in the OR as they are central components. Nevertheless, positional plausibility for OR tables must be ensured regarding safety and security [6], especially if other devices rely on the provided information. Examples are angiography systems, which enable image-guided surgery, or surgical robots. Both rely on the correctness of the determined OR table positions to avoid, e.g., collisions and thus severe patient harm. This, however, bears some unique challenges, as OR tables deform under a load of heavy patients, whereby the maximum patient load for a middle-class OR table is ∼250 kg (e.g., Getinge Maquet Meera [8]). The plausibility check in the sense of detecting anomalies in the measurement of sensor data, e.g., for position or load, using mathematical models for non-linear deformation is a challenge. In addition, the body, especially of heavy patients, deforms due to gravitational forces during movements of the OR table. This makes it also challenging to determine the actual physical values based on sensor measurements and detect possible errors in the system or its sensors without a high false alarm rate.

Problem: Anomaly detection in security systems primarily emphasizes routing-based attacks, with only a limited number of systems tackling physical signals like sensor data [9]. In addition, the mathematical model of a rigid body’s dynamic behavior is straightforward, but including the previously described deformation in such a model is not. One approach to overcome this obstacle is to use machine learning algorithms that can learn the nonlinear behavior of the deformation from training data. Let us assume that the system behavior and all occurring states can be written as a set $S=\{s_{0},s_{1},\ldots ,s_{n}\}$. The conventional states to be modeled via a physical system description can be written as $S=\{\int_{0},\int_{1},\ldots ,\int_{n}\}$. The following, therefore, applies S⊂S. If we now transfer this relationship to a singular state $s_{t}\in S$ in the specific moment *t*, we can rewrite the relation through an addition of a disturbance $d_{t}$ of our modeled system:

(1) $$s_{t}=\int_{t}+d_{t}$$

However, machine learning-based approaches bear the challenge of data acquisition in OR and healthcare settings, since conditions for proper data collection are not given [10]. Data-based models for the state estimation of sensor data may result in inadequate quality, particularly as sensor noise increases [11]. Integrating these approaches with conventional model-based estimation is a more promising area for research.

Contribution: The manipulation of deformable objects is a growing research problem in robotics, especially in healthcare applications such as surgical procedures [12]. Meanwhile, models representing the characteristics of the surgical robot and the human body can improve the safety of operations [13], for example, when checking the plausibility of measurements such as joint positions. To improve the sensing of deformable objects, Zhu et al. propose a hierarchical sensing model. It combines a simple model with a more sophisticated nonlinear model, such as a linear model as a base layer combined with a deep neural network that learns the rest of the behavior [12]. Such models can be built for OR tables to check the plausibility of position data. Based on intelligent sensor systems such as a load detection system [14] and a corresponding fault detection system [15], we suggest an anomaly detection approach for similar medical devices to contribute to a safer OR.

**Figure 1:**
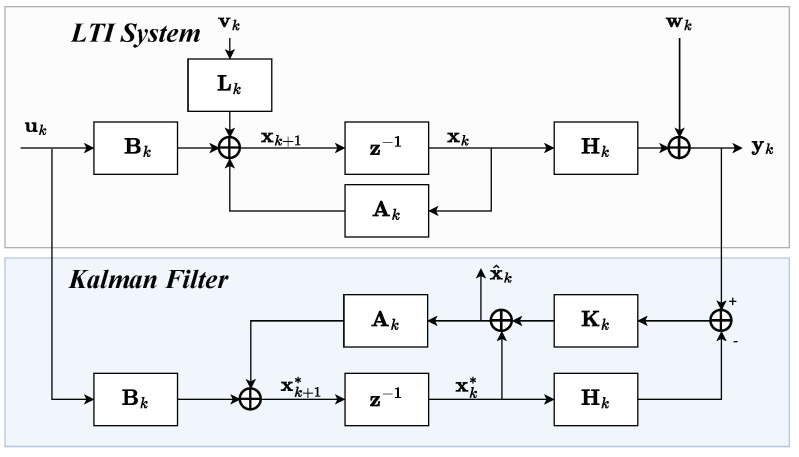
Signal processing for a stochastic disturbance-affected observed system using a KF [16] in a Time-Discrete Linear Time Invariant (LTI) system [17] with measurable input variables.

First, we propose a generic approach that uses and combines different anomaly detection techniques. Furthermore, we provide an example of a distributed software architecture to detect anomalies in an embedded environment. Our proposed system for anomaly detection involves a combined modeling approach. We use a Kalman Filter (KF) (Figure 1) to model the deductive system and patient behavior, while a data-based algorithm learns any indeterminable or non-linear behavior. *The advantage of this approach is that the algorithm does not have to learn and know the behavior. Instead, it only learns the misbehavior of the manual model, which is then used for anomaly detection.* This ensures that our system can detect anomalies that may not be accounted for by manual modeling.

Outline: Section 2 provides an overview of anomaly detection approaches using KFs, classical statistical methods Section 1, and machine learning methods. Section 3 will review related work in anomaly detection. The concept of applying hybrid filters using KFs as dynamic models in combination with data-based models for anomaly detection is presented in Section 4, followed by an evaluation of a prototypical implementation in Section 5. A discussion of the results and the effectiveness of the approach is conducted in Section 6. Finally, Section 7 provides a summary and suggestions for future work in this area.

## 2. Background in Anomaly Detection

Outlier data points can be described as observations that are so different from the other observations that it is suspected that they were generated by some other mechanism [18]. Anomaly detection techniques identify data points as such [19]. In this application, the working range of values of the fusion method used must be monitored to ensure that trustworthy values are generated. We have adapted the notation of formula symbols and equations to the standard notation used in the literature for algorithm notation.

### 2.1. Kalman Filter (KF)

Since our goal is to combine the KFs, which we generally refer to here as dynamic models, with machine learning-based algorithms, we focus on the comparison of the presented linear KF, Extended Kalman filter (EKF) and Unscented Kalman filter (UKF), which assume a Gaussian distribution. Other KF-based state estimators, such as the Particle KF and the Ensembled KF, which are used for non-Gaussian distributions [20], are not considered in this paper.

**Figure 2:**
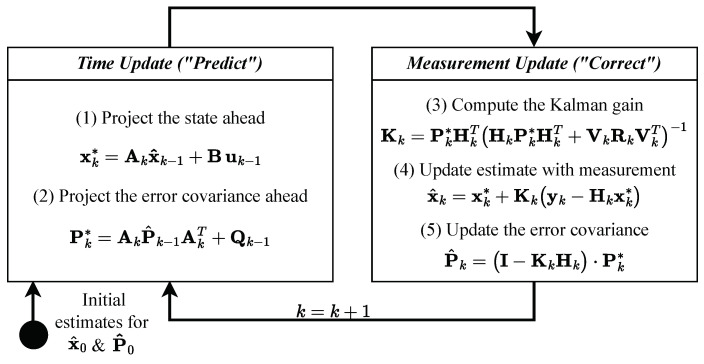
KF operation for each iteration *k* [22] (Table 1).

The KF [21] is a mathematical method that uses a process model in state space and a method for iterative state or signal estimation of time-independent signals in transient random processes [16]. In such processes, the mean and variance moments depend on time. This tool has been widely used in various fields, including aerospace, robotics, and finance, due to its ability to accurately and efficiently estimate the state of a system in the presence of noise and uncertainty. An iteration consists of a prediction step for the state x of dimension *m* (Table 1) that uses the state space model and a correction step that improves the prediction with a measurement y of dimension *n* (Figure 2). The KF is only suitable for a few variables in a linear system model but has a low computational cost [20]. When constructing the difference vector of the predictions or estimations of the KF with the measurements, a deviation score can be generated using $l_{k}$ norms. If the deviation exceeds a defined threshold value for an iteration *k*, the corresponding state or measurement vector of that iteration is classified as an anomaly. Thus, the KF is also a valuable approach to anomaly detection, e.g., when using the $l_{1}$ norm, which is also called the Mean Absolute Error (MAE):

(2) $$MAE_{state}=\frac{1}{n}\sum_{i=1}^{n}\left|{\hat{x}}_{i}-y_{i}\right|$$

**Figure 3:**
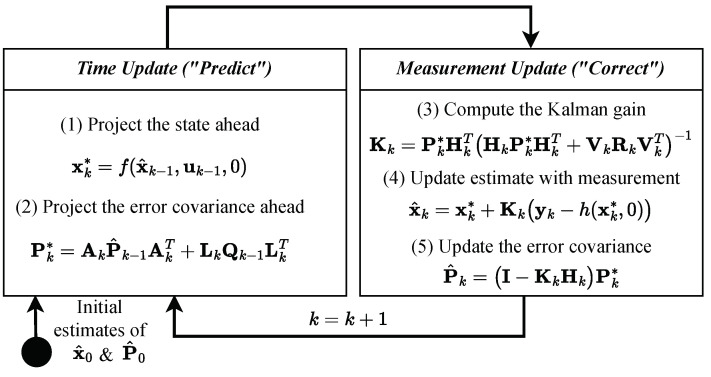
EKF operation for each iteration *k* [22] (Table 1).

The traditional KF is unsuitable for dealing with processes or measurements with nonlinearity. The EKF is used to overcome this limitation, approximating the nonlinear system by linearizing it around the current mean and covariance [22]. To estimate the current state of a system, the nonlinear state transition function $f({\hat{x}}_{k-1},u_{k-1},0)$ is used to predict the state. Similarly, the nonlinear observation function $h(x_{k}^{*},0)$ is used to project the estimated state $x^{*}$ onto the observation y (Figure 3). Although the EKF can be used for non-linear system models and has low computational costs, divergences may occur due to the linearization [20].

**Figure 4:**
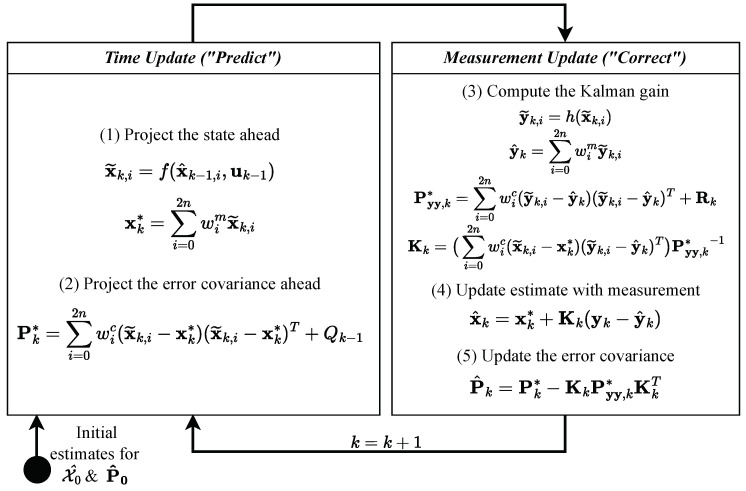
UKF operation for each iteration *k* [25] (Table 1 and Table 2).

For highly nonlinear problems, the UKF (Figure 4) [24] is generally more accurate than the EKF [25]. It uses the probability distribution of states by mapping so-called weighted sigma points through the nonlinear function and calculating the new mean and variance from this [20]. The UKF avoids the problems that can occur due to the linearization divergences of the EKF but requires more computational resources and performance degrades as the number of state variables increases [20].

The sigma points X for the state are calculated by a sigma-function $s(\hat{x},\hat{P})$. For the calculation of sigma points, the Van der Merwe Scaled Sigma Points are mainly used in industry and research as they represent a balanced compromise between accuracy and performance [25].

### 2.2. Statistical Methods

To check whether sensor measurements y values or the state estimation $\hat{x}$ of a KF are within the intended working range and plausible, statistical models can be used even in the most trivial case. This includes the Z-score, which has shown promising results in other applications [26]. The Z-score is defined as follows:

(3) $$Z=\frac{\left(x-\mu \right)}{\sigma }$$

Here, *x* is a single data point and a scalar, but for general validity we write $x_{i}$ as it is also possible to find multivariate inputs here. μ is the mean and σ is the standard deviation of $x_{i}$. The threshold value at which an anomaly is present can then be selected in the interval $W_{Z}=\left[2,3.5\right]$ depending on the application and data distribution. If data that are approximately normally distributed are available, the Z-score can be used as a relative metric. If this is not the case, the Inter Quartile Range (IQR) can instead be calculated as an absolute metric, which is also applied in outlier detection frameworks [27]. First, the IQR is calculated:

(4) $$IQR=x_{0.75}-x_{0.25}$$

Afterward, we define the interval of our common value distribution:

(5) $$I_{dist}=\left[\left(x_{0.25}-1.5\cdot IQR\right),\left(x_{0.75}+1.5\cdot IQR\right)\right]$$

The definition of the outlier set can then be written as $X_{out}\forall x∌I_{dist}$. The disadvantage becomes clear when the variables under consideration are no longer scalars, but vectors or even tensors: While there may still be strategies for some low-dimensional x, with $x_{i}\in R^{d}$ using Boolean algebra (and ∧/or ∨), this is hardly practical for images, audio signals or large sensor arrays where $x_{i}\in R^{D}$ with d<<D.

### 2.3. Machine Learning Based Methods

In addition to classical statistical methods, machine learning models can identify outliers. The advantage here is that the algorithms’ models draw their conclusions from the data provided and thus generalize better than, for example, purely statistical methods. This is particularly beneficial in real-world scenarios, where accurate knowledge of the data distribution is critical to the application of statistical methods. The Z-score, for example, requires normally distributed data, which is a condition that is rarely met in practice.

#### Unsupervised Learning

Since it is possible that there are no labels available to identify when the measurements or estimations of the KF are insufficient in the intended feature space, unsupervised learning is attractive here. A potentially suitable algorithm that meets our requirements is the Isolation Forest (IF), as it requires little memory, has a linear time complexity, and is not computationally expensive because no inter-distances and densities are calculated [28]. The conceptual idea behind this algorithm is that for any given $x_{i}$, a traversal is made from the leaves to the tree’s root, where the number of edges traversed is described by $l(x_{i})$. A recursive call is assumed to pass through a partition of the data points, where the length $l(x_{i})$ is a measure of the normality of the respective data point. Since this distribution is randomized between $x_{max}\in X$ and $x_{min}\in X$ where $X=\{x_{i}\in R|0<i<\infty \;,i\in N\}$, it can be assumed that extremely large or small values have shorter lengths. To determine a quantitative value of the anomaly, the use of normalization is desired, but this is not possible due to the different growth possibilities of the maximum length $l_{max}\left(x\right)=\left|X\right|$ and the average length of ${\bar{l}}_{avg}=log\left(|X|\right)$. However, since the isolation tree has an equivalent structure to the binary search tree, the average path length can be derived from it:

(6) c(|X|)=2H(|X|−1)−(2(|X|−1)/|X|)

Here, H(i) represents the harmonic number, which is uniquely described by the elements *i*, i.e., |X|, via the mathematical series of the harmonic sequence. The final anomaly score can be derived from this, as we can now use c(|X|) to normalize the path length $l(x_{i})$.

(7) $$s(|X|,x_{i})=2^{-\frac{E(l(x))}{c(|X|)}}$$

Here, E is the expectation value of a collection of isolation trees. The following anomaly values follow for the respective contexts [28]:

- E(l(x))→c(|X|),s(|X|,x)→0.5
- E(l(x))→0,s(|X|,x)→1
- E(l(x))→|X|−1,s(|X|,x)→0

Three possible scenarios can now be derived from this [28]:

- Data points very close to 1 are most likely anomalies.
- If all points are close to 0.5, the sample data set probably has no anomalies.
- If the values are clearly below 0.5, they are probably data points that can be classified as normal.

### 2.4. Deep Learning Based Approach

In principle, the use of neural networks is also suitable for anomaly detection, although a distinction must be made here between different model architectures. Nevertheless, the basic functionality of neural networks should be briefly outlined here. A neural network maps an input variable tensor to an output, which can be either a vector space or a discrete set. In general, the following function can be written for a neural network without restriction:

(8) $$N_{\Theta }:X\to \hat{Y}$$

Thus, it can be written: $N_{\Theta }:x_{i}\to \hat{y}$, where $x_{i}\in X\in R^{n}$ and $\hat{y}\in \hat{Y}$. This is to be understood abstractly, as Y can be a set, a subset, or a continuous value. $N_{\Theta }$ is parameterized via a set Θ. Precisely, these parameters can be found during training, which can be described as follows:

(9) $$\Delta \theta_{i,j}=-\alpha \frac{\partial L(\Theta ,x_{i},y_{i},{\hat{y}}_{i})}{\partial \theta_{i,j}}.$$

Therefore, it is necessary first to run a forward pass through the model to obtain the output ${\hat{y}}_{i}$, calculate an abstract cost function L using the true value, the label, $y_{i}$ and then find out how much the individual weights $\theta_{i,j}$ contribute to the change in the inference. The amount of the updating of the weights is determined by the learning rate α, e.g., to prevent overshooting and to get as close to the global minimum as possible. The aim is, therefore, to adjust the parameters Θ in such a way that the transmission function (see Equation (10)) of the model results in $\hat{y}\approx y$. This is the case if: $\Theta =\Theta^{*}$.

(10) $${\hat{y}}_{i}=\Gamma \left(\Theta^{*}x_{i}+b\right)$$

**Figure 5:**
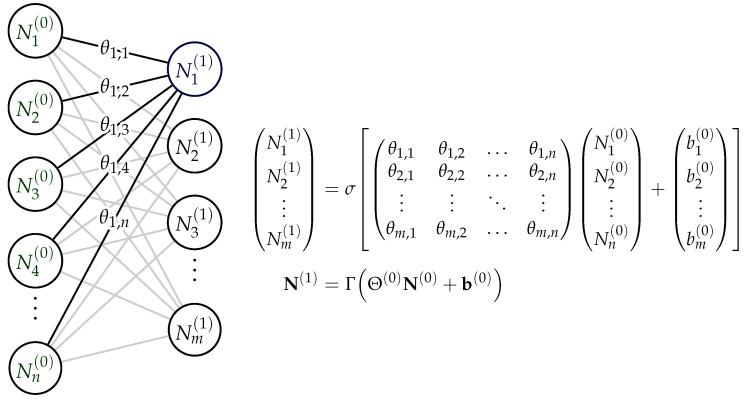
Basic neural network functionality with transmission function.

Here, Γ is a (mainly non-linear) activation function that allows the approximation of any continuous transfer function [29]. Figure 5 illustrates the principle and shows the transfer from one neuron in a layer to the next one [30]. Although deep learning has a high computational cost in the training phase, this approach will be analyzed in more detail here due to its power and adaptability.

#### 2.4.1. Autoencoder (AE)

**Figure 6:**
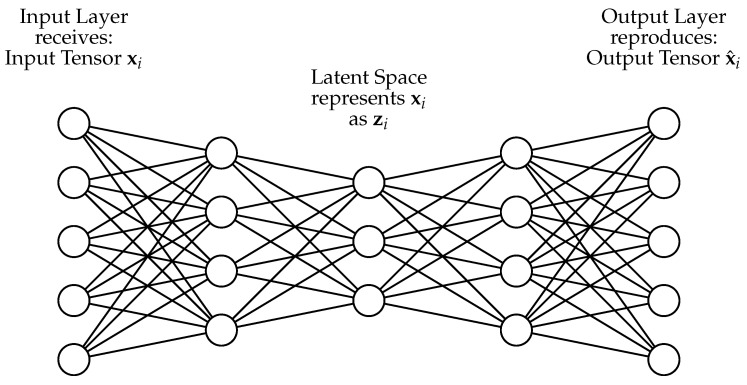
Functional principle of an AE.

Anomaly detection using Autoencoders (AEs) is sufficiently well researched, and now state of the art [31]. Here, the input features are mapped to themselves after the inference so that $f\left(x\right)\to \hat{x}$. The advantage of using deep learning-based methods is that they work with data of any dimension and with unstructured data such as images and audio signals. Figure 6 schematically outlines the concept of an AE.

Here, the model consists of two parts. First, the input vector $x_{i}$ of a dimension *n* is compressed to a latent variable $z_{i}$ of dimension *c*, which is part of the latent space Z (see Equation (11)).

(11) $$E_{\Theta_{E}}:X\to Z$$

This intermediate representation of the input is now used to build a reconstruction ${\hat{x}}_{i}$ of the original variable $x_{i}$ with dimensionality *n* (see Equation (12)), where $R^{c}<R^{n}$.

(12) $$D_{\Theta_{D}}:Z\to \hat{X}$$

Both are parameterized over different sets: $(\Theta_{E},\Theta_{D})\subseteq \Theta$. Instead of generating a representation model, such as in an IF, and comparing the occurring data over it, the data points are reduced to a smaller dimension *c*, where $R^{d}<R^{D}$ holds. The assumption is that anomalies behave differently after this compression than non-anomalous data. Subsequently, a reconstruction of the data to the original input $R^{n}$ dimension is performed. From this, the reconstruction error (see Equation (13)) can be calculated, which is small for known examples and high for anomalous samples due to inconsistencies [32]:

(13) $$L_{R}=\sum_{j=0}^{n}{\left(x_{i_{j}}-{\hat{x}}_{i_{j}}\right)}^{2}$$

The practical challenge is to find a suitable threshold to declare a reconstruction anomaly. This can be conducted by defining thresholds via the training data, e.g., via a desired percentile. This would involve looking at which values are below the 99th percentile [33]. The threshold can then be set above this. Alternatively, there are also approaches in which the most significant error occurring during training is saved and then set as a threshold [34]. The following function is used: a value is declared as an anomaly *M* depending on its reconstruction error $L_{R}$ and the set threshold *T*: 14).

(14) $$f\left(L_{R}\right)=\left\{\begin{array}{cc} \; & M,\;\;L_{R}<T\\ \; & M,\;\mathrm{else}\; \end{array}\right.$$

#### 2.4.2. Long Short Term Memory (LSTM) Networks

**Figure 7:**
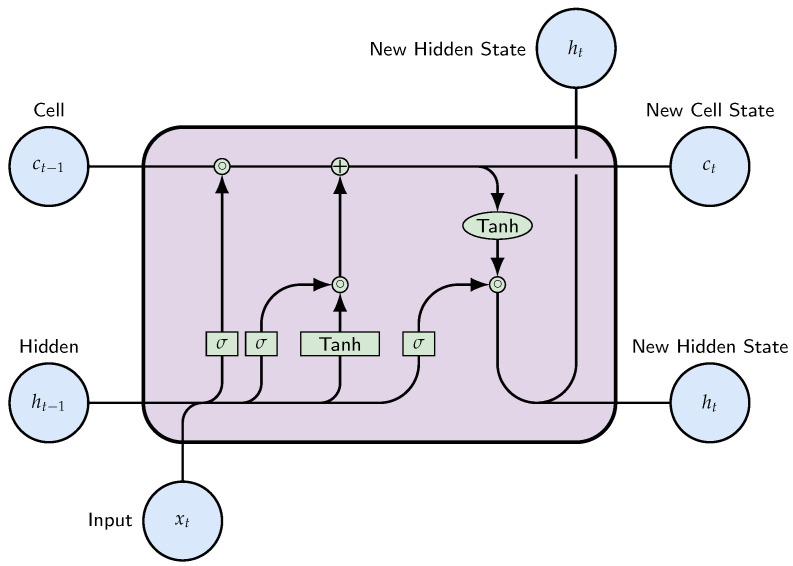
Structure of an LSTM-Cell.

A Long Short Term Memory (LSTM) is a Recurrent Neural Network (RNN) whose structure is shown in Figure 7. To solve the problem of the vanishing gradient, which can occur with such networks that are capable of processing transient time series data, the cell must store an internal state, the cell state $c_{t}$ [35]. The hidden state $h_{t}$ also exists. While the internal state $c_{t}$ is stored, the gates can adjust it successively. These gates, in turn, receive the state $h_{t-1}$ and the training data input. The forget gate decides which information is no longer needed in the cell state. To conduct this, the value $f_{t}$ is first calculated:

(15) $$f_{t}=\sigma \left(\Theta_{f}\cdot \left[h_{t-1},x_{t}\right]+b_{f}\right)$$

The forget gate activation function uses the sigmoid function to ensure that the output vector of the gate produces continuous values between 1 (for retention) and 0 (for forgetting).

(16) $$c_{f}=c_{t-1}\circ f_{t}$$

Next, the input gate is run through. Two functional units are are accommodated here. First, the direction of the adjustment is determined by hyperbolic tangent (W=[−1,1]), and then the amount of the adjustment is determined by a logical sigmoid operation, which is then used to calculate the influence on the internal state.

(17) $${\tilde{c}}_{t}=\tanh\left(\Theta_{c}\cdot \left[h_{t-1},x_{t}\right]+b_{c}\right)$$

(18) $$m_{t}=\sigma \left(\Theta_{m}\cdot \left[h_{t-1},x_{t}\right]+b_{m}\right)$$

The two values that have just been created are now processed together to update the internal state.

(19) $$c_{t}=c_{f}+m_{t}\circ {\tilde{c}}_{t}$$

Finally, the internal state is influenced by the output gate. Relevant information from the current and previous output is filtered here. The next output $h_{t}$ is then predicted. This is either the network output or serves as input for the next LSTM layer [36]:

(20) $$o_{t}=\sigma \left(\Theta_{o}\cdot \left[h_{t-1},x_{t}\right]+b_{o}\right)$$

(21) $$h_{t}=o_{t}\circ \tanh\left(c_{t}\right)$$

Such cells have a large number of parameters and are, therefore, challenging to train.

## 3. Related Work

Historically, most anomaly detection approaches have been model-based [37]. An anomaly detection process decides whether a value is considered an anomaly based on the deviation between the estimated and the measured result. Dynamic Bayesian Network (DBNs) are well-established for real-time anomaly detection in streaming data. These networks are dynamically expanded by incrementally adding new variables at each time step. New measurements are added according to a predefined template that outlines the conditional dependencies between features and their relationships with the existing network. The KF is an exemplary realization of a DBN in this context (Section 2.1). Hill et al. have effectively applied the KF to identify anomalies in low-resolution time series data [38,39]. However, such anomaly detection is highly dependent on the model’s accuracy. Model tuning is time-consuming and depends on prior knowledge of the system. Due to the limitations of model-based approaches, data-based approaches have received increasing interest.

A computationally inexpensive method is to check statistically whether a data point deviates from historical data. Statistical control charts [40], principal component analysis [41], and other statistical methods have been used.

With additional sensors and appropriate methods such as Fourier or Wavelet transforms, misbehavior can be detected, as shown in [42]. Due to the nature of classical signal analysis, only one-dimensional signals can be considered. Besides this limitation, additional sensors are only an option for some scenarios where space and cost must not be considered.

Machine learning-based methods are gaining attention due to promising results. In anomaly detection, a distinction can be made between supervised and unsupervised methods. Supervised methods, as shown in [43], are only suitable if a sufficient amount of labeled data is available.

In other domains, such as automotive in-vehicle communications, hybrid combinations of statistical, model-based, and machine learning-based anomaly detection have been explored to overcome the individual drawbacks of each method, as shown in [44]. Due to the underlying model, time series data can be processed to consider the temporal dependencies of anomalies. Another anomaly detection in the automotive domain is presented by Weber with the automotive observer [45]. This approach, which was initially developed for Controller Area Network (CAN) and later extended for Ethernet messages in automotive contexts in [46], have been adapted for general data analysis based on [44]. They use AEs and Lightweight On-line Detector of Anomalies (LODA) algorithms to detect signal anomalies, which have been modified to work with a sliding window for improved accuracy. The analysis is performed according to the ISO26262 standard, which ensures the reliability and consistency of the results. However, the authors do not incorporate dynamic models, they focus on single signals, and neglect the correlation of different signals. The authors use the *hybrid* term for the combination of specification-based anomaly detection and machine learning algorithms.

**Figure 8:**
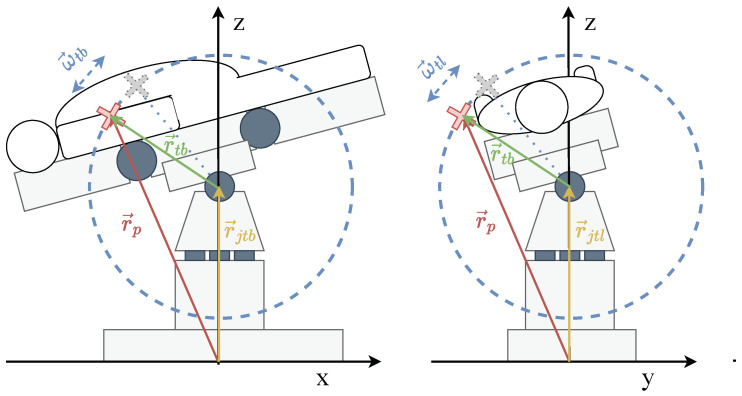
Overview of whole body movements Trendelenburg (**left**) and Tilt **(right)** used for the dynamic model (Table 3).

The error determination system presented in the patent [15] describes how a KF can determine sensor errors for a load recognition system for OR tables. Based on the prediction of the expected movement trajectory of the patient’s measured Center of Gravity (CoG) (Figure 8), the measurements are checked for plausibility. Nevertheless, the inventors do not recommend improvements in combining the KF with data-based models.

Huang et al. present a novel anomaly detection approach [47] specifically tailored for anomaly detection in time series data. The basic concept of their approach is to use the state estimation capabilities of the KF to improve the anomaly detection performance of an AE. This distinguishes their KalmanAE from traditional Kalman-based techniques, as it optimizes the KF using the embeddings produced by the AE. However, this method does not directly model the relationships between channels in multivariate time series data, an area that our approach aims to address.

In their study, Sanchez et al. [48] examine the robotic handling and detection of flexible objects and propose a novel categorization of these objects that takes into account not only their geometric form, but also their physical properties. In their analysis, they note that there is a significant gap in the field due to the lack of methods that provide universal solutions for different types of objects and tasks. In particular, they point out that the dynamic handling of flexible objects is still at an early stage of development.

In their paper, Cheng et al. present an unsupervised fault detection system for industrial robots based on a Gaussian mixture model using current signals [42]. They argue that the effectiveness of unsupervised machine learning algorithms in fault detection depends on the quality of the input fault features. They further propose that a better understanding of robotic systems through physics-based system analysis can help extract more effective fault features. The framework they propose involves pre-processing the signal and accurately distinguishing between different robot motion states in a measured current signal. Unique feature extraction is then used to derive robust fault features. These features are designed to be sensitive to faults but not to the robot’s movements. However, they do not address problems such as deformation.

The field of research into combining model-based and data-based methods, commonly called hybrid filters, has gained momentum in recent years. Jin et al. [11] have noted that this approach is beneficial when a system becomes challenging to model accurately and a promising way to enhance a model’s accuracy. One study by Liu et al. [49] demonstrated the effectiveness of this approach. They improved the predicted outcome of a model-based filter by training an LSTM model to estimate the discrepancy between the predicted and reference trajectories. By leveraging model-based and data-based approaches, hybrid filters can provide better results than using either method alone. However, they do not use their approaches to detect anomalies, a gap we target in this paper (Section 1).

## 4. Concept

Applying statistical methods (Section 2.2) that assume normal distribution to non-normal patient data will result in unwanted outliers since humans vary heavily in body proportions. The shoulder width, for example, varied from 1.5 to 3.25 head lengths for male subjects as examined by Kilgore [50]. This means that the standard deviation for the Z-score must be set so high to avoid false positives that the detection of true positives is unlikely. Even non-normally based methods such as IQR assume a probability distribution that does not adapt to individual patients. In addition, the static interpretability and generalization ability of, e.g., multivariate Z-scores become challenging, especially for high-dimensional data. In addition, statistical and information-theoretical methods are not suitable for modeling sequential data [51].

**Figure 9:**
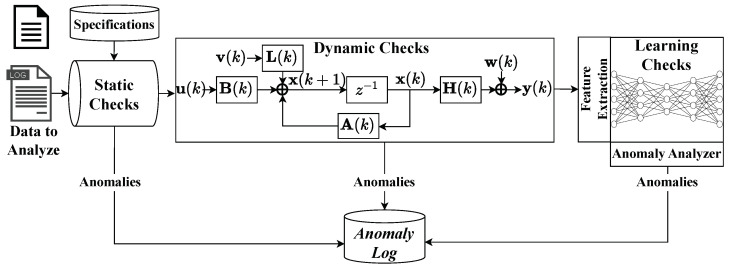
Extended anomaly observer concept based on [44].

Based on the automotive observer by Weber et al., which uses different types of plausibility checks to monitor anomalies (Section 3), we present a generic approach for anomaly detection systems using static, dynamic, and learning checks (Figure 9). Dynamic checks use dynamic models (Section 2.1) and learning checks use data-based models (Section 2.3 and Section 2.4). This paper focuses on combining the dynamic and learning checks since the dynamic checks are our proposed extensions, which are expected to reduce the number of features required (curse of dimensionality [30]) and still reduce the False Positive Rate (FPR). By using expert knowledge for the dynamic tests, the explainability given for a KF is also given for the hybrid variant. This means that only the part of the data-based learning check cannot be easily explained.

Accordingly, we have chosen a hybrid filter approach: The first part of the equation is represented by a Kalman filter KF to predict physically explainable system behavior. Here, the filter is parameterized through A,B,Q,H,R,L, (see Table 1) and receives an input tuple U,Y (see Table 1) to predict the state.

(22) $$KF_{\left(A,B,Q,H,R,L\right)}:\left(U,Y\right)\to \hat{X}$$

In the case of UKF/EKF the functions *h* and *f* replace A,H, and for UKF the sigma function *s* is an additional parameter. The machine learning model N only has to model $d_{t}$ from D in order to describe the system behavior that is physically difficult to model:

(23) $$N_{\Theta }:\left(\hat{X}-Y\right)\to D$$

Static checks are derivable from the system specification, leading to rules, e.g., that a velocity signal needs to be in a specified value range. Dynamic Checks are manually created physical models that are mathematically described as proposed here for the partial body movements of a patient (Section 4.1) or for whole body motions, as proposed in [15] (Section 3). Learning checks are data-based models such as AE, LSTM model (Section 2.4) or IF (Section 2.3) trained on data collected for the system. Each check can be combined and can provide input to the following checks at the same time. If one of the checks detects an anomaly, it is logged for further processing, such as user notification.

### 4.1. Dynamic Check for Partial Movements of the Patient’s Body

**Figure 10:**
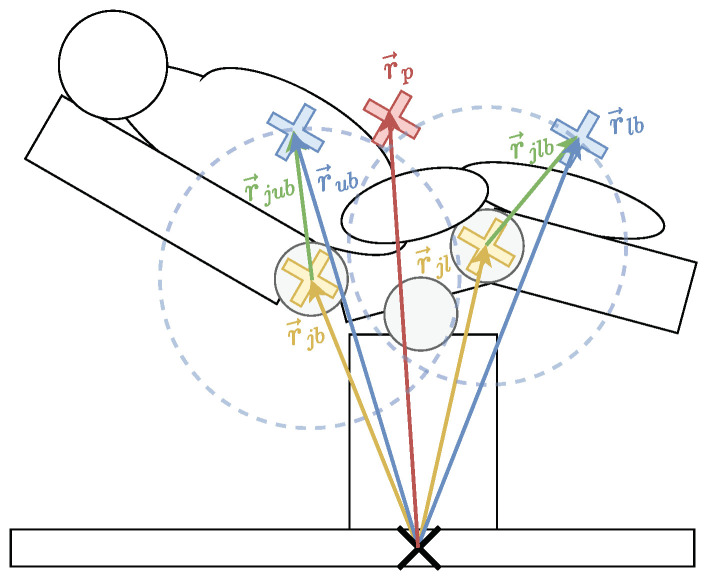
Patient position ${\vec{r}}_{p}$ in dependency to upper body distance ${\vec{r}}_{ub}$, lower body distance ${\vec{r}}_{lb}$, back joint distance ${\vec{r}}_{jb}$ and leg joints distance ${\vec{r}}_{jl}$ as well as the resulting distances ${\vec{r}}_{jlb}$ and ${\vec{r}}_{jub}$ describing the rotational distances (Table 4).

Given an OR table with a load determination system (Section 1), the overall CoG of the load can be determined. Therefore, the idea is to check the trajectories of the CoG during a movement for plausibility as described in [15] including movements of the patient’s body parts (Figure 10). Nevertheless, as a simplification, the dynamic check assumes that the patient’s body mass distribution and flexible elements are neglected, so rigid body dynamics can be assumed (Section 1).

In addition, for a proof of concept, it is sufficient to sum individual body parts into two big parts: the upper and lower body of the patient. This simplification allows a more straightforward calculation of the patient’s CoG. Specifically, for rigid body dynamics with homogeneous mass distribution, the patient’s upper body CoG position ${\vec{r}}_{ub}$ as well as the patient’s lower body CoG ${\vec{r}}_{lb}$ can be used to calculate the patient’s CoG ${\vec{r}}_{p}$.

To calculate the patient’s CoG, it is necessary to use a proportion $\lambda_{ub}$ for the moved upper body part with the back joint and a proportion $\lambda_{lb}$ for the impacted upper body part with the leg joint.

Neglecting the inhomogeneous mass distribution and deformation of the human body, the dynamics for rigid bodies can be applied to describe the position and velocity of the patient’s CoG:

(24) $${\vec{r}}_{p}=\lambda_{ub}{\vec{r}}_{ub}+\lambda_{lb}{\vec{r}}_{lb}$$

(25) $${\dot{\vec{r}}}_{p}={\vec{v}}_{p}=\lambda_{ub}{\vec{v}}_{ub}+\lambda_{lb}{\vec{v}}_{lb}=\lambda_{ub}{\vec{\omega }}_{jb}\times {\vec{r}}_{jub}+\lambda_{lb}{\vec{\omega }}_{jl}\times {\vec{r}}_{jlb}$$

To simplify matters, no tilting of the patient is considered, so only the rotation around the y-axis is considered for the leg and back joint, resulting in the y-axis value being irrelevant.

(26) $$\left[\begin{array}{c} r_{px}\\ r_{py}\\ r_{pz} \end{array}]=[\begin{array}{c} 0\\ \lambda_{ub}\omega_{jb}\\ 0 \end{array}]\times [\begin{array}{c} r_{jubx}\\ r_{juby}\\ r_{jubz} \end{array}]+[\begin{array}{c} 0\\ \lambda_{lb}\omega_{jl}\\ 0 \end{array}]\times [\begin{array}{c} r_{jlbx}\\ r_{jlby}\\ r_{jlbz} \end{array}\right]$$

(27) $$\left[\dot{\begin{array}{c} r_{ub,x}\\ r_{ub,z}\\ r_{lb,x}\\ r_{lb,z}\\ \omega_{jb}\\ \omega_{jl} \end{array}}]=[\begin{array}{c} \omega_{jb}\left(r_{ub,z}-r_{jb,z}\right)\\ -\omega_{jb}\left(r_{ub,x}-r_{jb,x}\right)\\ \omega_{jl}\left(r_{lb,z}-r_{jl,z}\right)\\ -\omega_{jl}\left(r_{lb,x}-r_{jl,x}\right)\\ 0\\ 0 \end{array}\right]$$

Other joints of the OR table are neglected in this scenario so that the positions of the joints ${\vec{r}}_{jb}$ and ${\vec{r}}_{jl}$ can be assumed as constants. Thus, they do not need to be measured and do not need to be contained in the state vector $\vec{x}$. Another simplification to reduce the dimension of the state is achieved by using a constant velocity model rather than a constant acceleration model (${\dot{r}}_{ub,x}=v_{ub,x}=C$). The non-linear measurement function h(x) can be calculated as follows:

(28) $$h\left(x\right)=[\begin{array}{c} \lambda_{ub}r_{ub,x}+\lambda_{lb}r_{lb,x}\\ \lambda_{ub}r_{ub,z}+\lambda_{lb}r_{lb,z}\\ \omega_{jb}\\ \omega_{jl} \end{array}]$$

### 4.2. Learning and Hybrid Filter Checks

Since the dynamic model is based on the assumptions of the standardized patient and a rigid OR table model with Gaussian process and measurement noise, the system will detect measurements with patients outside these norms as anomalies. Therefore, classical methods are expected to fail in these situations.

Thus, AE, LSTM model (Section 2.4), and IF (Section 2.3) are considered as standalone variants as well as hybrid variants in combination with the dynamic check for partial patient body movements (Section 4.1).

**Figure 11:**
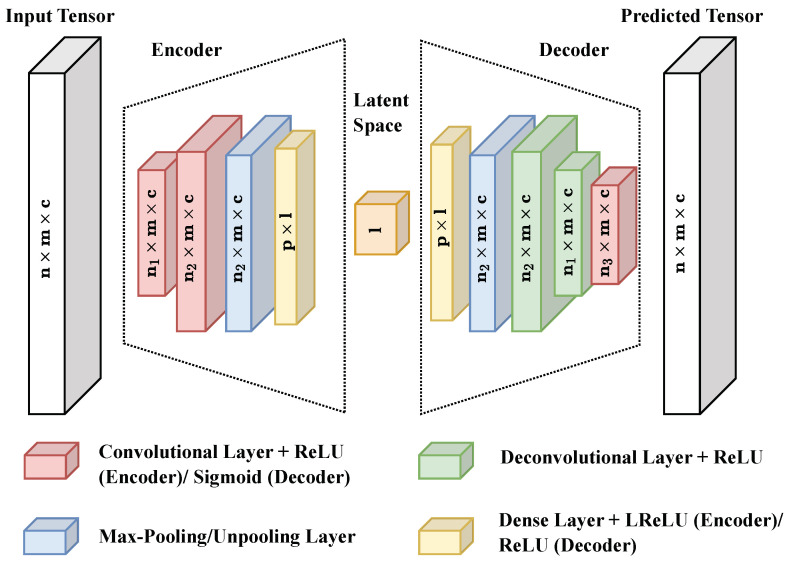
AE architecture (*n*: Number of windows, *m*: window size, *c*: Number of channels/features, $n_{1}$: Neuron number of first conv. and second deconv. layer, $n_{2}$: Neuron number of second conv. and first deconv. layer, $n_{3}$: Neuron number of third conv. layer, *p*: pooling layer size, *l*: latent space dimension).

It is common practice to use 1D Convolutional Neural Network (1DCNN) AEs (Figure 11) for analyzing time series data [52]. This design is also considered suitable for the scenario examined here. The encoder consists of two convolutional layers: a max-pooling layer and a dense layer. On the other hand, the decoder has a structure that is the mirror image of the encoder, which includes a dense layer followed by an unpooling layer and two deconvolutional layers. Finally, the decoder ends with another convolutional layer. The activation functions used are Rectified Linear Unit (ReLU), Leaky Rectified Linear Unit (LReLU), and Sigmoid.

**Figure 12:**
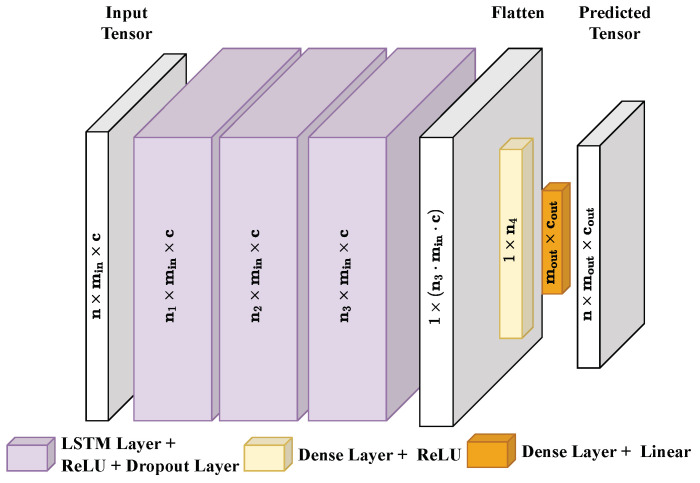
Implemented LSTM architecture with n: number of windows, $m_{in}$: window size, *c*: number of channels/features of input, $n_{i}$: neurons number of layer i, $m_{out}$: window size of predicted output tensor, $c_{out}$: number of channels/features of output.

The LSTM network model used in this study consists of three layers of LSTM units, with a dropout layer following each LSTM layer to prevent overfitting (Figure 12). The activation functions can be adjusted accordingly: Each LSTM layer contains a ReLU activation function. In contrast, a linear activation function should be used in the dense layer at the output. Also, other activation functions are usable, such as Swish activation instead of ReLU in the LSTM layers. However, the first approach was selected to predict the absolute expected error in this case.

## 5. Prototypical Implementation and Evaluation

In the following, we apply the generic approach (Section 4) and the safety system for detecting errors in load recognition systems of OR tables (Section 3) to monitor the influence of the back movement of an OR table on the patient’s CoG.

### 5.1. Data synthesis Using Digital Twins

**Figure 13:**
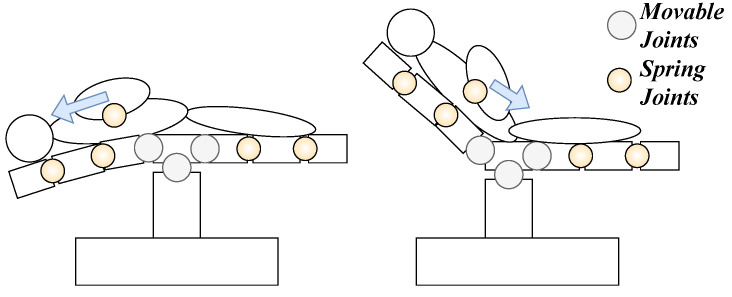
Patient and OR table models during a back movement.

In the context of medical robotics, simulation models are becoming increasingly prevalent for developing and testing surgical procedures and devices. A digital twin of the Gazebo OR table (Figure 13) is employed with Robot Operating System (ROS) 2 nodes for generating physical data, allowing the evaluation of algorithms under various scenarios. Simulation models offer a cost-effective and efficient approach to system development, minimizing the risk associated with physical prototypes and providing accurate representations of the system’s behavior and dynamics.

Flexible elements such as muscles and tissues need to be accurately represented to enhance the accuracy and reliability of patient models. Spring joints are applied here to model these elements, allowing a more realistic simulation of the patient’s body movement and deformation (Figure 13). The use of spring joints also enables the representation of the flexibility of the OR table’s components, which is crucial for accurate simulations of the system’s dynamics. These deformations are not designed into the dynamic model (Section 4.1) and, therefore, are expected to impact its estimation negatively.

**Figure 14:**
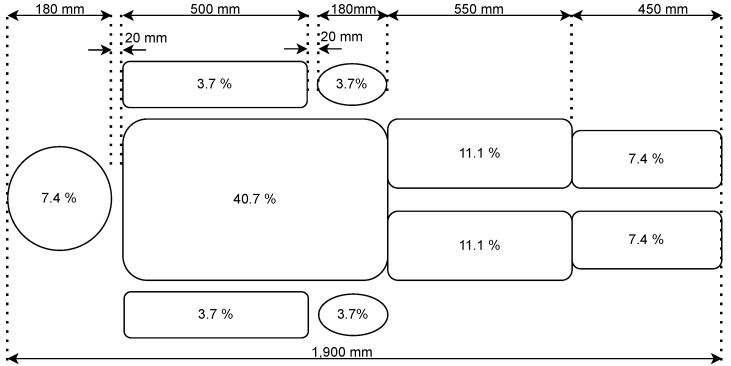
Patient mass distribution according to IEC60601-1 [53].

The patient model is created in Unified Robot Description Format (URDF) based on relevant standards and regulations in medical technology. Here, the IEC 60601-1 [53] is used as a foundation (Figure 14). Flexible elements are introduced at the abdomen and upper legs for heavy patients to represent the patient’s movement and deformation.

### 5.2. Distributed Anomaly Detection

**Figure 15:**
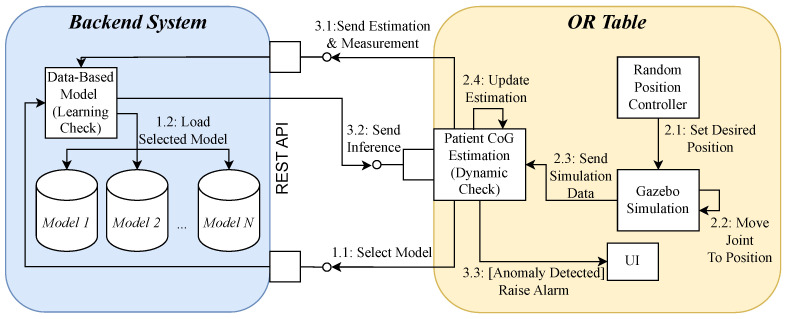
Communication diagram for distributed hybrid anomaly detection implementation.

Our architecture for hybrid anomaly detection consists of the OR table and a backend system (Figure 15). This server-client separation allows the computationally intensive data-based models to be processed in a cloud. In addition, flexibility is created as models can be exchanged or retrained without interfering with the embedded system of the OR table. The OR table uses a service-oriented architecture with Data Distribution Service (DDS) as middleware through the use of ROS2. The test setup consists of a controller service that represents the user interaction of the OR table. During simulation time, the OR table is moved to different target positions at random times.

Based on the Gazebo proxy data, a dynamic check of the patient CoG estimate is performed every 100 ṁs. By transferring the estimate and the measurement data to the backend system, the estimate is checked there for anomalies using data-based models. The results are logged in a Comma-Separated Values (CSV) file. The dynamic check of the CoG estimate is realized as an ROS2 node (see Section 5.4). Together with the learning checks (see Section 5.5–Section 5.7), the system forms the hybrid filter. In the case of a connection failure, the system performs a simplified independent check based on the ROS2 node alone.

The connection between the backend system and the OR table is established over a REpresentational State Transfer (REST) interface. Several REST services provide the interaction needed for the dynamic checks and the simulation environment. At startup, the trained models are selected over a REST interface based on the currently determined patient weight and preferred data-based model (LSTM, IF, or AE) and loaded into the learning check. With another ROS2 node, random positions are commanded to the Gazebo OR table simulation that executes the desired joint movement. This is then sent to the *Patient CoG Estimation* dynamic check using the ROS1/ROS2 Bridge.

The dynamic check estimates each patient’s CoG, which is then sent to the backend system for evaluation. The inference is sent back to the node for the patient estimation. If the estimated value is an anomaly, the UI raises an alarm triggered by the dynamic check.

In a real-world OR scenario, we consider the backend system either as part of the hospital IT, as a dedicated cloud server provided by the device manufacturer, or as a supervision system directly in the OR, as proposed in [54]. In the latter scenario, the OR table would send the estimation data to the OR monitoring system via a SDC network, which has more computational resources than the embedded system of the OR table. The dynamic check is considered to be executable on a performant embedded system and thus, for example, as part of a dedicated communication gateway of the OR table, comparable to the architecture proposed in [7].

### 5.3. Classification of Deviations as Anomaly

As mentioned in Section 2.4, a threshold must be found for the AE and the LSTM network used to ensure a clean categorization of the anomaly definition. These include:

- The reconstruction error of the AE variants
- The LSTM prediction of the difference value of the KF output to the measurement
- The absolute difference of the pure LSTM position prediction to the measurements

When using the IF, the grouping is based on the path length and thus implicitly finds its threshold (Section 2.3).

Similar to [33], we have decided to use the 99th percentile. There are several reasons for this method over a manual threshold, such as the ability to adapt to new data and the creation of objective comparability between the approaches in the evaluation. The choice of percentile can also be changed. However, a low FPR is vital for our medical application as false alarms in hospitals [55] lead to alarm fatigue, causing critical alarms to be missed [56], and reducing the quality of care [57]. Accordingly, we have selected a correspondingly high percentile to meet this target. In addition, each signal, e.g., CoG in x and z direction, is evaluated separately using a dedicated threshold. Therefore, an input tensor is classified as an anomaly when it exceeds the threshold value in one of these signals.

**Figure 16:**
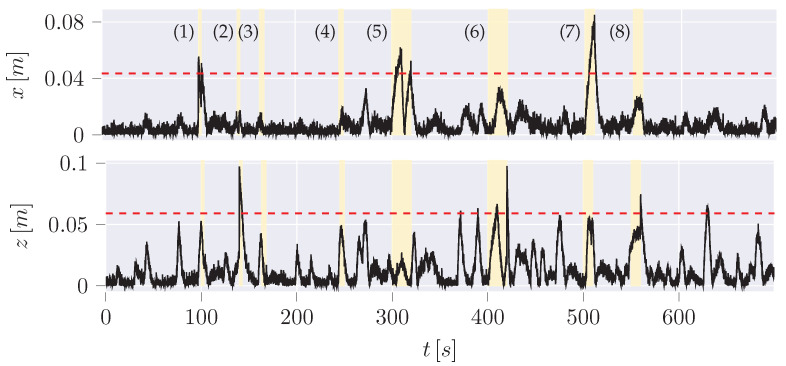
Absolute error of the estimated CoG by the UKF to the measurement data including anomalies (yellow highlighted area, Table 5).

Four types of anomalies, each in the x and z position (Figure 16), are used for the evaluation of the different algorithms based on the overview of anomalies by Weber [45] (Table 5).

### 5.4. Partial Body Movement UKF

As a dynamic check for partial body movements, the UKF and EKF algorithms have been implemented. These implementations are structured as ROS2 nodes, which subscribe to the positions and velocities of the CoG derived from the Gazebo simulation. The estimated CoG positions and OR table joint velocities are logged into a CSV file for comprehensive analysis and training of the data-based models. A dedicated internal class, Gazebo Proxy, facilitates communication between the Gazebo environment and the dynamic checks. The estimations are updated every 100 milliseconds to balance real-time responsiveness and computational efficiency. However, as the results are similar for the simulated scenario, only the UKF is discussed here for further examination because the EKF is expected to create similar results as its hybrid variant.

With the previously mentioned simplifications (Section 4.1), the patient’s body is only divided in the lower and upper body. Thus, according to the standard patient of IEC60601-1 [53], the upper body is considered to be $\lambda_{ub}=0.63$, and the lower body is considered to be $\lambda_{lb}=0.37$. When a patient is seated in a beach chair position (Figure 10), which matches the scenario under examination, their upper body makes up around 63% of their overall mass.

### 5.5. Isolation Forest (IF)

**Figure 17:**
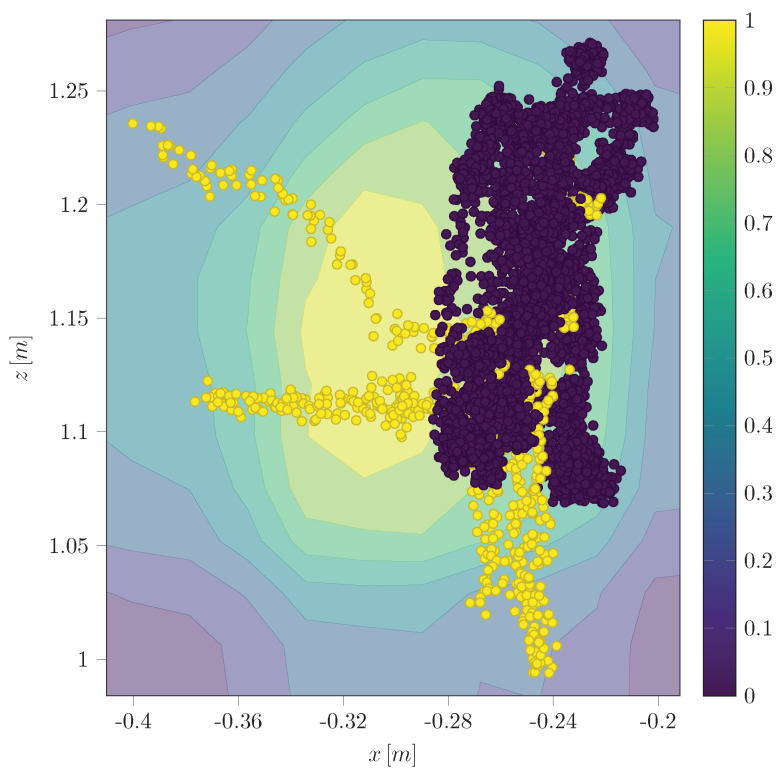
Anomalies (yellow) detected by the pure IF in position measurement x and z.

**Figure 18:**
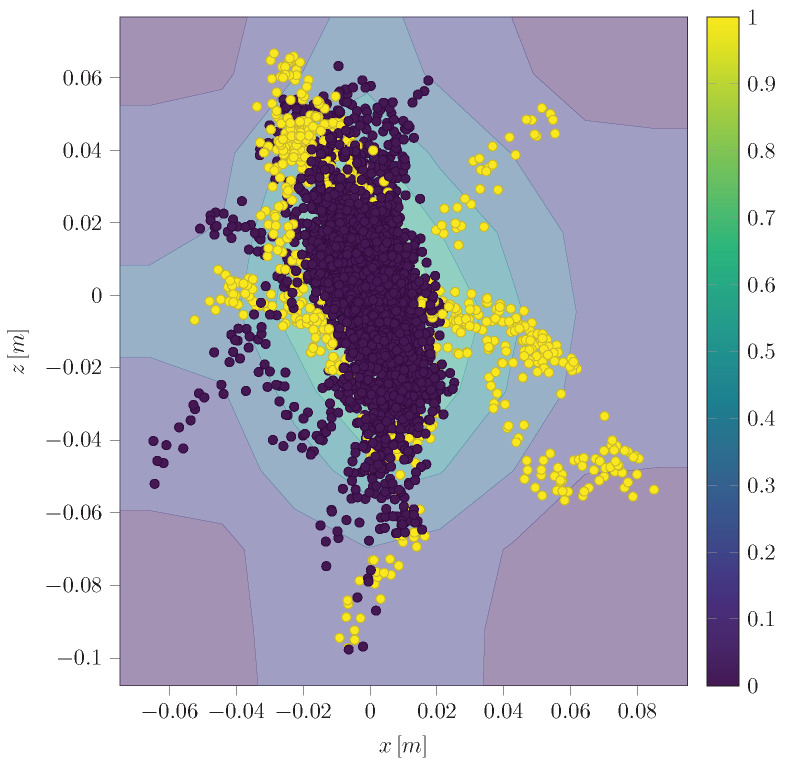
Anomalies (yellow) detected by the hybrid IF in difference of UKF estimation to the measurement of positions.

The Python implementation of our IF utilizes the Scikit-learn [58] library and its dependencies. Our implementation can take dynamic model estimations, sensor measurements, or their difference as inputs. The standalone variant is trained on the position measurements (Figure 17). On the other hand, the hybrid variant of the implementation uses a two-dimensional input of the difference between the measurement and the UKF estimation in the x and z CoG direction (Figure 18).

We do not select any time window to ensure the IF implementation is minimal, and evaluate the low-resource variant of the hybrid filter. As the IF does not require a manually predefined threshold for classification into anomalies (Section 2.3), the training data primarily affect the evaluation metrics such as FPR and True Positive Rate (TPR), and there is limited manual influence for subsequent fine-tuning in inference time.

In our result, the pure IF (Figure 17) is not capable of determining meaningful thresholds, leading to high FPR (∼40%) and False Negative Rate (FNR) (∼55.5%). At the same time, the hybrid variant centers the normal data in between the thresholds so that the FPR (∼13%) as well as the FNR (∼18.8%) can be improved significantly.

**Figure 19:**
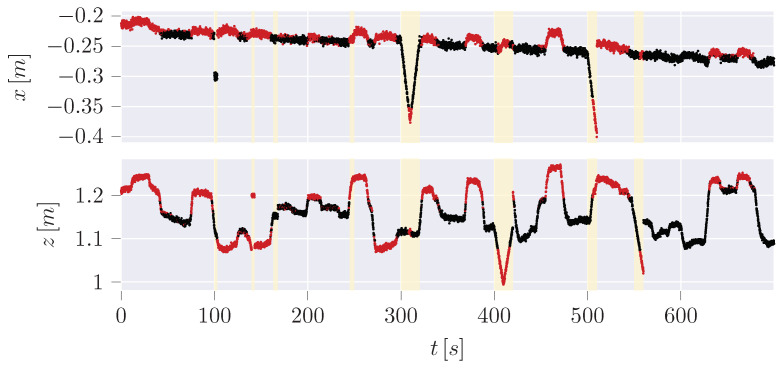
Detected anomalies (red) in time series for IF.

**Figure 20:**
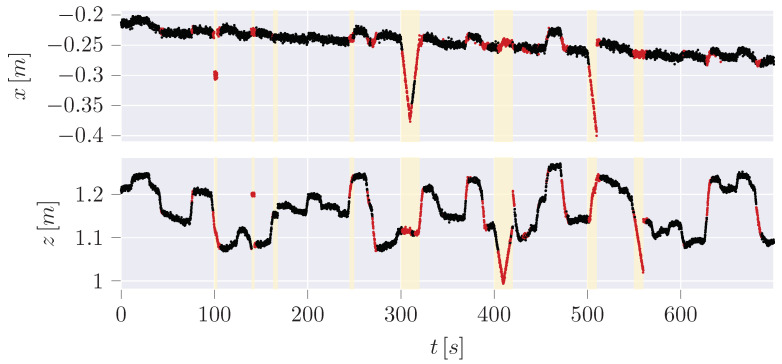
Detected anomalies (red) in time series for hybrid IF.

Since the trajectories of anomalies can be guessed from the hybrid plot, a disadvantage of the hybrid variant or at least of the classification of anomalies is revealed: The anomalies are classified based on the manipulation of the measurement data. It is not considered that the dynamic model, which is the UKF here, may need time to recover from a sudden change of the measurement after an anomaly back to normal data. Especially in jump scenarios, the difference between measurement and estimation will remain high for a short period until the dynamic model has recovered. These data points are not classified as anomalies here, which will also increase the FNR of all examined learning checks. However, it is noticeable in the time series of the pure IF (Figure 19) that most of the detected anomalies do not correlate with the anomalies in contrast to the hybrid variant (Figure 20).

### 5.6. LSTM

The LSTM network is realized in Python using the TensorFlow library [59]. It is suitable for various feature combinations for predicting the output based on the position and joint velocity measurements and the estimated state of the KFs (Section 4.1), which can be arbitrarily combined, including a variable time series.

**Figure 21:**
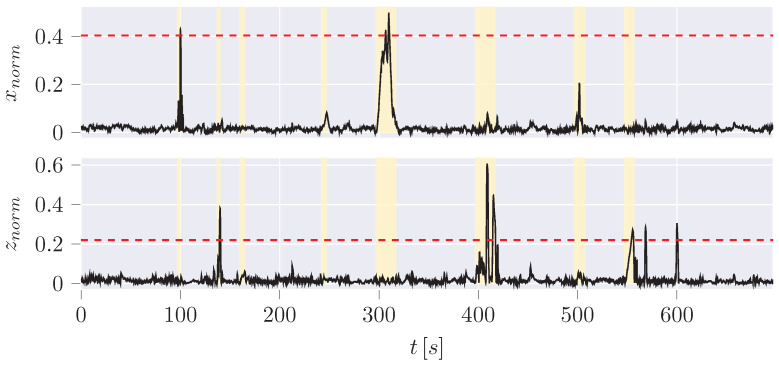
Absolute scaled difference between estimation and measurement to forecast at LSTM.

As the LSTM predicts the next value (Figure 21), we consider the pure variant as a deep learning variant to the dynamic model. Therefore, we chose all position and velocity measurements as input for a time window of 30 values, which corresponds to 3 s. However, the LSTM does have a disadvantage compared to the UKF, which is not iteratively updated based on all previous measurements. Instead, it uses an adequate time window, which needs to be exploratively determined.

**Figure 22:**
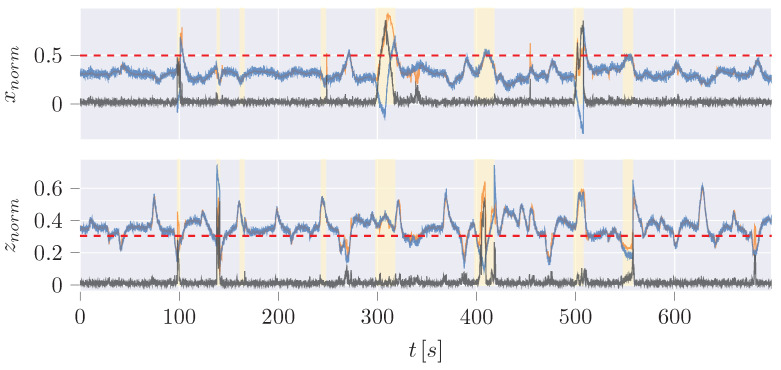
Difference (black) of the absolute error (difference of estimation and measurement for CoG position *x* and *z*) of the UKF (blue) predicted by an LSTM (orange).

The hybrid LSTM variant is designed to predict the estimated deviation of the following UKF iteration based on a time window of 20 values corresponding to 2 s. The error of the UKF estimation compared to the measurement (blue) (Figure 22) is, therefore, directly predicted by an LSTM (orange). The absolute difference is then used as the basis for the anomaly determination (black).

The best results are achieved with the hybrid LSTM in terms of FPR as it decreases the FPR of the standalone LSTM by a factor of 30 (∼0.6% to ∼0.02%), while also decreasing the FNR by nearly 14%. In both cases, this leads to high precision. Furthermore, it is conspicuous that both LSTM variants have a high threshold based on the 99th percentile compared to the average non-anomaly data or even compared to peaks in the non-anomaly data. Other architectures of recurrent neural networks, such as GRU, will behave in a similar way to LSTM, which is why a closer look can be omitted at this point. They can also lead to serious problems such as vanishing gradients (vanilla LSTM) and are, therefore, considered obsolete.

### 5.7. Autoencoder (AE)

**Figure 23:**
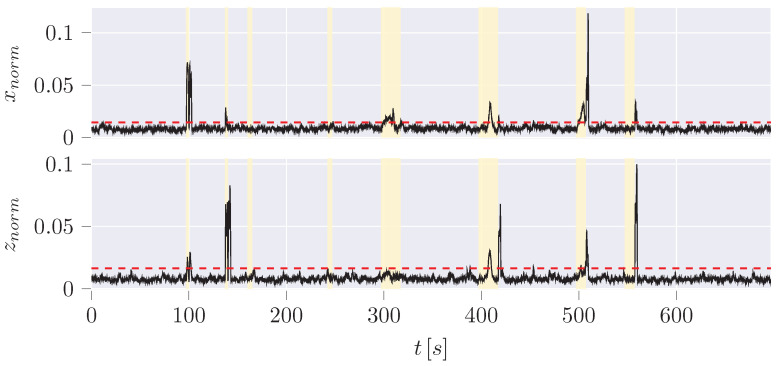
AE reconstruction MAE for position difference of estimation to measurement x and z.

**Figure 24:**
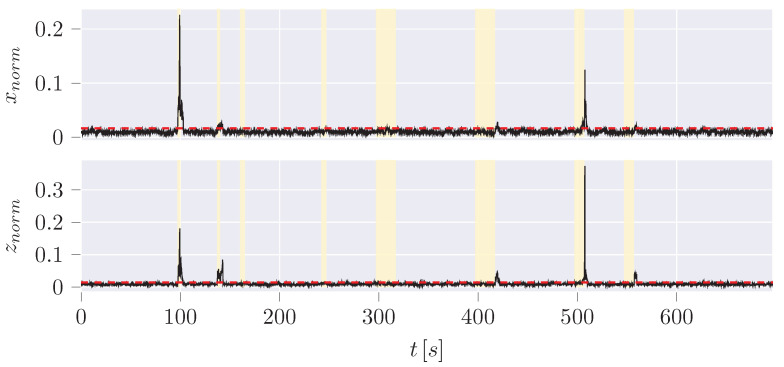
First hybrid AE variant reconstruction MAE for position difference of estimation to measurement x and z.

The PyTorch library in Python is used to implement the AE, which is used to reconstruct a desired time series of estimations and measurements [60]. The reconstructed CoG x and z values in a time window of 30 values (3 s) are generated by the basic AE, (Figure 23). The first hybrid variant, on the other hand, employs the difference between UKF estimation and measurement using the same window size (Figure 24).

We calculate the reconstruction error for all variants as MAE, meaning that the average of all reconstructed values compared to the measurement is calculated as follows:

(29) $$MAE_{window}=\frac{1}{n}\sum_{i=1}^{n}\left|{\hat{x}}_{i}-x_{i}\right|$$

where

- *n*: Number of data points in the window.
- ${\hat{x}}_{i}$: Reconstructed value for the *i*-th data point.
- $x_{i}$: Measured value for the *i*-th data point.

**Figure 25:**
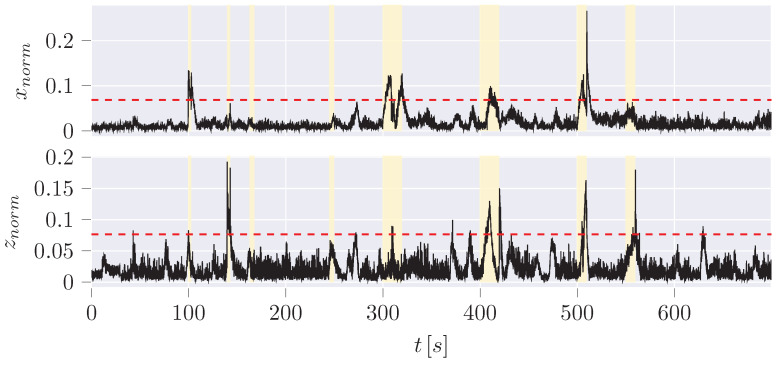
Second hybrid AE variant reconstruction MAE of measurements with window size 4 reconstructing UKF estimations and measurements of positions.

While the AE has a higher FPR (2.3%) and FNR (65.5%) with smaller window sizes, another approach to the hybrid AE reveals improved performance compared to the difference between dynamic model estimation and measurement (FPR: 2.7%; FNR: 87.9%): When using the measurements and estimations as separate values, a time window of four results in a lower FPR and FNR (Figure 25), while increasing the window size worsens the results. In addition, the latent space can be reduced from size 8 to 2 and improve the performance at the same time.

The hybrid AE shows that although the FPR and FNR cannot be lowered for the examined scenario, the second approach shows that training effort and window size can be decreased, which makes this approach more interesting for higher dimensional tasks. It slightly increases the FPR by 1%, but decreases the FNR by 16.3% compared to the UKF.

## 6. Discussion

### 6.1. Discussion on Pure Learning & Dynamic Checks

Table 6 compares the confusion matrices of all data-based algorithms with the UKF. The pure LSTM network performs best for true-negative and false-positive, closely followed by the UKF. Although the pure IF performs best on false-negative and true-negative data, the difference between correctly and incorrectly classified cases is so insignificant that the data point only has a circa 58.4% chance of being classified correctly (accuracy). Hence, the practical relevance of this approach is questionable.

Table 7 compares the metrics of all examined data-based algorithms and the UKF as representative for the dynamic checks. While the pure LSTM fulfills the aim of a low false-alarm rate the most (FPR of 0.6%), the other methods outperform it in the other metrics. The UKF has the highest precision (72.6%), the AE has the highest accuracy (90.8%), the highest F1 Score (45.1%), and the highest ROC AUC Score (66.1%). Therefore, for a balanced anomaly detection performance, the AE is the best choice for a pure dynamic/learning check. As previously stated, the practical relevance of the pure IF performance is questionable, although it has the highest recall (44.5%) and the lowest FNR.

### 6.2. Discussion on Hybrid Filters

Table 8 compares the confusion matrices of the different hybrid filters. Here, the hybrid LSTM network performs best in the case of true-negatives and false-positives, while the hybrid IF performs best in the case of false-negatives and true-negatives. The hybrid AEs offers a solution between the performance of the others, while the second variant is significantly more promising.

Table 9 compares the metrics of all examined hybrid filters. Overall, the hybrid LSTM network fulfills the aim of low false alarms (Section 5.3) best since it has the highest accuracy, precision, and lowest FPR. However, severe anomalies might not be identified with the high FNR and low recall. Here, AE and IF in both model variants perform better.

Also, in the case of the hybrid IF, the FPR and the FNR can both be significantly improved by a factor of approximately 3. Still, it is much higher than the LSTM models. Nevertheless, the hybrid IF is the best choice if a balanced FPR and FNR is desired with high recall and F1 Score. All algorithms except for the hybrid IF, which is not evaluated based on the 99th percentile, have a high FNR of more than 50%. *Thus, we assume that the chosen anomalies represent hard-to-find ones* due to partially minor deviations in the measurement compared to the true values that are additionally covered by noise. Therefore, we do not reach the F1 value of 0.7 typically targeted in the literature, and in addition, the 99th percentile is a very conservative threshold. However, the jump anomalies (1 & 2) are detected by all checks. In comparison, the stuck-at anomalies (3 & 4) are only discovered in the x (3) direction by the AEs, but only shortly above the threshold.

Therefore, it is likely that an ideal anomaly detection system, as proposed in Section 4, uses a mixture of checks to cover as not all seem to be discoverable with all algorithms. In addition, the FPR could be reduced further by a voting system of several checks, e.g., an anomaly must be discovered in at least two checks. Furthermore, only the LSTM variants outperform the dynamic check implemented as UKF in terms of the FPR. Nevertheless, all data-based and hybrid models outperform the UKF in terms of FNR and recall.

## 7. Conclusions and Future Work

Regarding feature reduction and possible data combination, whether the pure database models can keep their performance is questionable. More features may result in less distinguishable anomalies (distance in multidimensional space). Therefore, reducing necessary features and a smaller window size of the hybrid filters may outperform the entire setup for position monitoring of the patient. Furthermore, in all cases, the hybrid variant improves performance. In addition, for the AE and LSTM cases, the feature set, including the necessary window size, can be reduced compared to pure data-based models.

An application-specific advantage of the proposed check is that the position of the patient represented as CoG results from the entire kinematic chain and thus the entire OR table joint positions. Checking the patient’s CoG is sufficient to check all other joint positions for plausibility. If one of these positions is determined incorrectly, this leads to deviations in the movements. For example, suppose the patient is tilted around their longitudinal axis. In that case, there will also be a change in the y-axis during a movement of the upper body, which is not predicted by the dynamic model (Section 4.1) and would, therefore, lead to deviations.

The scenario chosen for evaluation is only a small subset of the capabilities of the OR table and the possibilities of patient positioning and anthropometry. The data collected via a simulation of 5 h of driving the back joint in a static scenario is enough to yield results on the performance of several algorithms. It is questionable if all the scenarios not examined here can be covered with the data collected in the field. While in the automotive industry manufacturers can reach out to the data of millions of cars increasing year by year, in the medical device industry, such as for the OR table, manufacturers can only use the data of few hundred to a thousand each year. In addition, not all of these OR table are used for all patient types, positions, and surgical procedures [7].

The primary focus to improve the presented hybrid anomaly detection lies in selecting an appropriate data-based model and design, as this choice substantially impacts the results. Following this, optimizing the dynamic model becomes the next priority. The lower priority is the enhancement or expansion of the training dataset, serving as the final adjustment to further refine overall performance.

As the hybrid AEs, in general, needs smaller windows and especially the IF could be improved significantly using the UKF estimation, we assume that most of the historical information is stored in the state of the KF. Therefore, also other internal states of the KF that result from sensor fusion and are not measured, e.g., ${\vec{r}}_{ub}$ or ${\vec{r}}_{lb}$, can additionally improve the results. Thus, we expect the performance of the hybrid filters to be heavily dependent on the design of the dynamic model.

As this paper concentrates on a single device, the approach can be applied to interoperable medical devices in an SDC network (compare with the approach presented in [54]). The different devices in the network may observe each other, creating a more robust system of systems. Each medical device in the OR is supervised by backend features, and compromising a single heavily connected device might be more challenging than a standalone system with minimal connectivity interfaces. However, this also requires analyses of response times, especially when hard real-time is needed.

To enhance the outcome, it is possible to assess other KFs that utilize Interacting Multiple Model (IMM) algorithms to adjust to varying patient positions and types. Moreover, since the second variation of the hybrid AE employs a small window, a DenseAutoencoder may be more appropriate than the currently selected 1DCNN AE.

The current estimation could be corrected to the likely value as another possible next step to recover from anomalies. This can be conducted with the values predicted from the LSTM or the AE’s reconstructed window. This could also increase the availability of the medical device in case of anomalies, vastly improving safety.

In the case of a safety or security incident, it is mostly not important which data point was an anomaly, but only the fact that an anomaly has occurred for several seconds or even minutes and with high confidence (small FPR). Therefore, the uncertainty should be calculated and logged, e.g., using Monte Carlo Sampling.

Finally, a comprehensive runtime and resource consumption evaluation must be carried out in the form of a benchmark for the various combinations of algorithms, also in comparison to their pure variants. This also includes the runtime evaluation for distributed approaches using a backend system. However, this requires a more extensive prototype implementation in a distributed architecture under hospital network conditions.

## Figures and Tables

**Table 1:** KF variables [16]. Since v is expected to be white noise, L can be neglected [23].

Variable	Description
$x^{*(m)}$	Predicted system state vector
${\hat{x}}^{\left(m\right)}$	Estimated system state vector after new observation y
$u^{\left(l\right)}$	System input vector
$y^{\left(n\right)}$	New observation/measurement vector
$B^{\left(m\times l\right)}$	Dynamics of the system input u and projection on system vector x
$K^{\left(m\times n\right)}$	Kalman-Gain-Matrix to project the residuals onto the correction of the system state
$A^{\left(m\times m\right)}$	Transition-Matrix to propagate the system state to next time point
$P^{*\;(m\times m)}$	A-priori covariance matrix of the predicted system state before new observation y
${\hat{P}}^{\left(m\times m\right)}$	A-posteriori covariance matrix of the estimated system state after new observation y
$Q^{\left(m\times m\right)}$	Process noise covariance matrix for uncertainties from modeling errors or changing boundaries
$H^{\left(n\times m\right)}$	Observation matrix projecting the system states on the measurement
$R^{\left(n\times n\right)}$	Covariance matrix of the measurement noise
L	Input matrix projecting the input noise v on the system state $x^{*}$

**Table 2:** UKF variable overview addendum to Table 1.

Variable	Description
${\tilde{x}}_{i}$	*i*-th sigma point of the predicted state
X	Set of *i* sigma points ${\tilde{x}}_{i}$ of the predicted state
${\tilde{y}}_{i}$	*i*-th sigma point of the predicted measurement
${\hat{y}}_{k}$	Predicted measurement
$w_{i}^{m}$&$w_{i}^{c}$	Sigma point weights for mean and covariance calculation

**Table 3:** Overview of variables for whole body movements.

Variable	Description
${\vec{r}}_{p}$	Position of the patient’s CoG in world coordinates
${\vec{v}}_{p}$	Velocity vector of the patient’s CoG
${\vec{\tilde{\omega }}}_{tb}$	Angular velocity vector of the rotational Trendelenburg joint in world coordinates
${\vec{\tilde{\omega }}}_{tl}$	Angular velocity vector of the rotational tilt joint in world coordinates
${\vec{r}}_{tl}$	Distance vector between the tilt joint origin and the CoG of the patient (${\vec{r}}_{p}-{\vec{r}}_{jtl}$)
${\vec{r}}_{tb}$	Distance vector between the trend joint origin and the CoG of the patient (${\vec{r}}_{p}-{\vec{r}}_{jtb}$)

**Table 4:** Overview of variables for partial body movements.

Variable	Description
${\vec{r}}_{p}$	Position of the patient’s CoG in world coordinates
${\vec{v}}_{p}$	Velocity vector of the patient’s CoG
${\vec{r}}_{ub}$	Position of the patient’s upper body in world coordinates
${\vec{r}}_{lb}$	Position of the patient’s lower body in world coordinates
${\vec{r}}_{jb}$	Position of the back joint origin
${\vec{r}}_{jl}$	Position of the leg joint origin
${\vec{\tilde{\omega }}}_{jb}$	Angular velocity vector of the rotational back joint in world coordinates
${\vec{\tilde{\omega }}}_{jl}$	Angular velocity vector of the rotational leg joint in world coordinates
${\vec{r}}_{jub}$	Distance vector between the back joint origin and the CoG of the patient’s upper body (${\vec{r}}_{ub}-{\vec{r}}_{jb}$)
${\vec{r}}_{jlb}$	Distance vector between the leg joint origin and the CoG of the patient’s lower body (${\vec{r}}_{lb}-{\vec{r}}_{jl}$)

**Table 5:** Position anomaly scenarios (Figure 16).

Anomaly	X-axis	Z-axis	Potential Scenario
Positive/Negative Step Plateau	(1) jump to (x = −0.3 m) between 100 and 103 s (3 s)	(2) jump to z=1.2m between 140 and 143 s (3 s)	Indicative of a sensor defect or a potential attack where specific positions are set.
Plateau	(4) *x* stuck at a value between 245 and 250 s (5 s)	(3) *z* stuck at a value between 163 and 168 s (5 s)	A defect or manipulation attempt that keeps the current position value stuck at the current value while recovering the actual value after the anomaly.
Positive/Negative Ramp Plateau	(5) *x* increase from 300–310 s (10 s), decrease from 320 to 330 s with ≈1.33cm/s	(6) *z* increase between 400 and 410 s (10 s), decrease from 410 to 420 s with ≈1.33cm/s	Indicates a possible sensor error or an attempt by an attacker to manipulate the position to a desired value gradually.
Positive/Negative Ramp with Jump Back	(7) *x* increase between 500 and 510 s (10 s) with ≈1.33cm/s	(8) *z* increase between 550 and 560 s (10 s) with ≈1.33cm/s	Indicates a possible sensor error or an attempt by an attacker to manipulate the position to a desired value gradually, ending with a jump to the actual value.

**Table 6:** Confusion matrices of pure data-based and dynamic models.

Model	AE		IF		LSTM		UKF	
	False	True	False	True	False	True	False	True
**False**	6064	141	3746	2488	**6167**	**37**	6169	65
**True**	502	264	**425**	**341**	674	92	594	172

**Table 7:** Metrics of all pure learning and dynamic checks.

Metric	AE	IF	LSTM	UKF
**Accuracy**	**0.908**	0.584	0.898	0.906
**Precision**	0.652	0.121	0.713	**0.726**
**Recall**	0.344	**0.445**	0.120	0.225
**F 1 Score**	**0.451**	0.190	0.205	0.343
**ROC AUC Score**	**0.661**	0.523	0.557	0.607
**False Positive Rate**	0.023	0.400	**0.006**	0.010
**False Negative Rate**	0.655	**0.555**	0.880	0.775

**Table 8:** Confusion matrices of hybrid filters in comparison.

Hybrid Model	AE (Variant 1 & 2)		IF		LSTM	
	False	True	False	True	False	True
**False**	6040/6107	165/124	5424	810	**6213**	**1**
**True**	673/469	93/297	**144**	**622**	571	195

**Table 9:** Hybrid filter metrics in comparison in all anomaly scenarios and normal behavior data.

Metric	Hybrid AE	Hybrid IF	Hybrid LSTM
**Accuracy**	0.880/0.915	0.864	**0.918**
**Precision**	0.360/0.705	0.434	**0.995**
**Recall**	0.121/0.388	**0.812**	0.255
**F 1 Score**	0.182/0.500	**0.565**	0.405
**ROC AUC Score**	0.547/0.684	**0.841**	0.627
**False Positive Rate**	0.027/0.020	0.130	**0.0002**
**False Negative Rate**	0.879/0.612	**0.188**	0.745

## Data Availability

Data is contained within the article.

## Abbreviations

The following abbreviations are used in this manuscript:

1DCNN	1D Convolutional Neural Network
AE	Autoencoder
CAN	Controller Area Network
CSV	Comma-Separated Values
CoG	Center of Gravity
DBN	Dynamic Bayesian Network
DDS	Data Distribution Service
EKF	Extended Kalman filter
FNR	False Negative Rate
FPR	False Positive Rate
IF	Isolation Forest
IMM	Interacting Multiple Model
IQR	Inter Quartile Range
KF	Kalman Filter
LODA	Lightweight On-line Detector of Anomalies
LReLU	Leaky Rectified Linear Unit
LSTM	Long Short Term Memory
LTI	Linear Time Invariant
MAE	Mean Absolute Error
OR Table	Operating Room Table
OR	Operating Room
REST	REpresentational State Transfer
RNN	Recurrent Neural Network
ROS	Robot Operating System
ReLU	Rectified Linear Unit
SDC	Service-oriented Device Connectivity
TPR	True Positive Rate
UKF	Unscented Kalman filter
URDF	Unified Robot Description Format
